# Non-invasive diagnosis of wheat stripe rust progression using hyperspectral reflectance

**DOI:** 10.3389/fpls.2024.1429879

**Published:** 2024-09-11

**Authors:** James F. Cross, Nicolas Cobo, Darren T. Drewry

**Affiliations:** ^1^ Department of Food, Agricultural, and Biological Engineering, Ohio State University, Columbus, OH, United States; ^2^ Environmental Sciences Graduate Program, Ohio State University, Columbus, OH, United States; ^3^ Facultad de Ciencias Agropecuarias y Medioambiente, Universidad de La Frontera, Temuco, Chile; ^4^ Department of Horticulture and Crop Science, Ohio State University, Columbus, OH, United States; ^5^ Translational Data Analytics Institute, Ohio State University, Columbus, OH, United States

**Keywords:** wheat stripe rust, hyperspectral reflectance, remote sensing, machine learning, random forest, dimensionality reduction

## Abstract

Wheat stripe rust (WSR), a fungal disease capable of inflicting severe crop loss, threatens most of global wheat production. Breeding for genetic resistance is the primary defense against stripe rust infection. Further development of rust-resistant wheat varieties depends on the ability to accurately and rapidly quantify rust resilience. In this study we demonstrate the ability of visible through shortwave infrared reflectance spectroscopy to effectively provide high-throughput classification of wheat stripe rust severity and identify important spectral regions for classification accuracy. Random forest models were developed using both leaf-level and canopy-level hyperspectral reflectance observations collected across a breeding population that was scored for WSR severity using 10 and 5 severity classes, respectively. The models were able to accurately diagnose scored disease severity class across these fine scoring scales between 45-52% of the time, which improved to 79-96% accuracy when allowing scores to be off-by-one. The canopy-level model demonstrated higher accuracy and distinct spectral characteristics relative to the leaf-level models, pointing to the use of this technology for field-scale monitoring. Leaf-level model performance was strong despite clear variation in scoring conducted between wheat growth stages. Two approaches to reduce predictor and model complexity, principal component dimensionality reduction and backward feature elimination, were applied here. Both approaches demonstrated that model classification skill could remain high while simplifying high-dimensional hyperspectral reflectance predictors, with parsimonious models having approximately 10 unique components or wavebands. Through the use of a high-resolution infection severity scoring methodology this study provides one of the most rigorous tests of the use of hyperspectral reflectance observations for WSR classification. We demonstrate that machine learning in combination with a few carefully-selected wavebands can be leveraged for precision remote monitoring and management of WSR to limit crop damage and to aid in the selection of resilient germplasm in breeding programs.

## 1 Introduction

Stripe rust, primarily affecting cereals such as wheat, rye, barley, and various grass species, is one of the most severe and widespread plant diseases globally (Chen et al., 2002; Wellings, 2011; Figueroa et al., 2018). Wheat stripe rust (WSR) is caused by *Puccinia striiformis* f. sp. *tritici* (*Pst*), an airborne fungal pathogen capable of transmitting over extensive distances and resulting in total crop loss in severe cases (Waqar et al., 2018). Characteristically stripe rust manifests through the formation of yellow to orange stripes on the leaves, leaf sheaths, glumes, and awns of susceptible plants (Chen et al., 2014). Like many rust fungi, *Pst* is an obligate biotrophic parasite that absorbs nutrients and water from living tissue (McIntosh et al., 1995; Lin et al., 2018; Chen, 2020). Stripe rust can cause up to 100% yield loss in susceptible cultivars, especially when the disease starts early and continues to develop during the growing season (Chen, 2005). An estimated 88% of global wheat production is susceptible to *Pst*, threatening the wheat industry as a whole (Schirrmann et al., 2021). In 2021, the United States Department of Agriculture (USDA) reported a 5.9 million bushel loss of wheat due to wheat stripe rust, highlighting the economic and agricultural impact of this disease (Kolmer and Fajolu, 2021). The appearance of new highly aggressive *Pst* races with broader virulence profiles and tolerance to high temperature (Milus et al., 2009; Hovmøller et al., 2015) have prompted the expansion of *Pst* epidemics to warmer areas (Hovmøller et al., 2011). The long-range dispersal and rapid evolution of these new races (Hovmøller et al., 2008; Milus et al., 2009; Ali et al., 2014) have brought about a rapid erosion of effective resistance genes, dramatically reducing the number of effective sources of resistance available for breeders to protect new varieties (Lowe et al., 2015).

Integrated management strategies that combine genetic resistance and crop management can help mitigate the effects of the disease (Beres et al., 2020). Early detection is crucial for effective *Pst* management, to prevent spore production and dispersal, but also reduce fungicide usage overall (Moshou et al., 2004; Carmona et al., 2020; Prahl et al., 2022). Modern fungicides represent a convenient alternative to control wheat rusts, though their application adds a significant cost to production (Chen, 2005; Chen et al., 2014) and may lead to health and environmental risks when not used properly (Cobo et al., 2018). Breeding resistant varieties to replace those susceptible to new *Pst* races is the most effective, economic, and environmentally friendly way to control current stripe rust epidemics (Hovmøller et al., 2010; Liu et al., 2017; Cobo et al., 2019; Zhou et al., 2021) and prevent their further expansion (Cao et al., 2012). Developing genetic resistance has been at the forefront of efforts to reduce the threat of stripe rust globally (Singh et al., 2005; Chen, 2020). However, this strategy requires permanent efforts to identify and deploy new sources of resistance against the rapidly evolving *Pst* populations (Cobo et al., 2018; Zhou et al., 2021). The identification of genes associated with stripe rust resistance, and the type and strength of resistance, requires field evaluations of segregating populations that have been inoculated to promote strong and even infection (Cobo et al., 2018; Qiao et al., 2024). Remote sensing offers tremendous potential to provide accurate, non-invasive and repeatable assessments of plant disease status and resistance (Nilsson, 1995; Mahlein, 2016), particularly as advances in imaging technologies and machine learning converge (Arsenovic et al., 2019; Saleem et al., 2019; Sishodia et al., 2020; Weiss et al., 2020; Schirrmann et al., 2021).

The timely and reliable discovery and characterization of new sources of resistance to highly virulent *Pst* races and the continued advancement of genetic resistance will depend on new capabilities to detect and quantify stripe rust through high-throughput techniques (Schirrmann et al., 2021), ideally providing objective and repeatable assessments of the response of plants to the pathogen to allow for more precise selection of resistant genotypes. Feature detection in imagery has proven to be a powerful technique for plant disease detection in general (Saleem et al., 2019), and WSR detection specifically (Azadbakht et al., 2019; Schirrmann et al., 2021), but requires high-resolution imagery and sufficient lighting conditions to produce reliable and reproducible results. Visible through shortwave infrared (VSWIR) spectroscopy, often referred to as hyperspectral sensing or imaging spectroscopy, provides a rich source of information on a variety of plant biophysical traits, e.g. water, pigment and nutrient contents (Ustin et al., 2004; Goetz, 2009; Kokaly et al., 2009; Ustin et al., 2009; Krishna et al., 2014; Asner et al., 2015). Hyperspectral VSWIR sensing offers significant potential to advance plant disease detection and rating through detection of changes to plant biophysical traits impacted by disease, rather than image analysis (Mahlein et al., 2018). Terentev et al. (2022) recognize the capabilities of hyperspectral sensing for early plant disease detection before symptoms are visible to human observers or typical RGB cameras. The latent period in WSR, the time from first infection to the appearance of symptoms, can be 10-14 days under ideal conditions (Murray, 2005). Early detection of WSR would allow commercial producers to take advantage of early acting treatments, reducing overall costs and preventing further disease spread (Carmona et al., 2020). Automation of disease monitoring methods promises to expand the capabilities of wheat producers to protect their fields but has met with several challenges on the quantification of disease severity and risk (Ashourloo et al., 2016; Shafi et al., 2022, 2023).

Prior work on wheat disease monitoring has primarily focused on disease detection and severity assessment through measurements of the diseased percentage of leaf coverage (Wang et al., 2007; Zhang et al., 2014; Ashourloo et al., 2014a, b; Yao et al., 2019; Maqsood et al., 2021; Jiang et al., 2023; Zhao et al., 2023). In this study, we use a modified 10-class severity scale (Peterson et al., 1948) which is designed to better capture early symptoms of disease infection providing a rigorous basis for breeders to evaluate WSR resistance in new accessions. This large number of finely resolved classes provides a unique challenge for our assessment of the ability of hyperspectral reflectance and machine learning to classify WSR severity.

Here we assess the ability of the information contained in hyperspectral VSWIR sensing to effectively classify WSR disease severity at both the leaf and canopy in two susceptible varieties with different stages of infection. We used random forests as the machine learning framework, along with dimensionality reduction approaches to produce efficient models that demonstrate significant skill in disease severity identification. Feature importance is used to identify the specific spectral regions that are most important at both leaf and canopy scales. This work provides a path to effective utilization of hyperspectral VSWIR reflectance for the automated scoring of disease severity in breeding programs and will likewise facilitate timely precision treatment applications in production contexts to maximize the efficiency of anti-fungal treatments at field-scale.

## 2 Materials and methods

### 2.1 Experimental design

**Figure 1:**
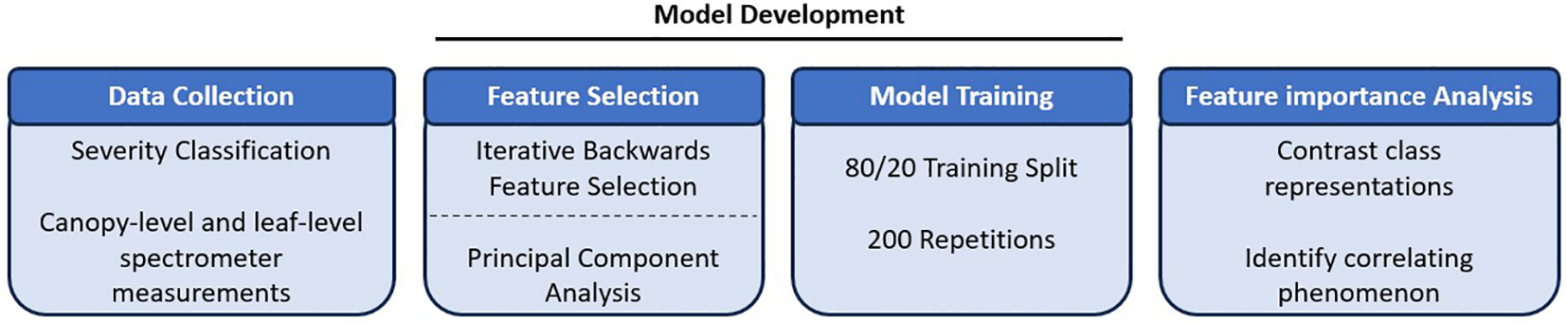
Schematic of the experiment and analytical process.

**Figure 1** provides a schematic of the analytical process used in this experiment. Leaf samples and reflectance spectra were collected from two susceptible cultivars with a range of stages of *Pst* infection. Leaf and canopy-level hyperspectral reflectance samples were collected across the range of rust infection spanning a 10-class and 5-class severity classification scale, respectively. Random forests was used to examine the ability of reflectance across the 450-2400nm spectral range to classify stripe rust severity across these fine scales typical of breeding population evaluations. Model performance and feature importance were quantified. The sub-sections below describe each component of this process in greater detail.

### 2.2 Study site and plant material

Field experiments were initiated in mid-November at the University of California field station near Davis, California (38°31” N, 121°46” W) in a Yolo loam soil (fine-silty, mixed, superactive, nonacid, thermic Mollic Xerofluvents). Fertilization consisted of 224 kg N ha^−1^ applied as (NH_4_)_2_SO_4_, half at pre-planting and the rest at the beginning of jointing.

Highly susceptible common wheat lines ‘DS6301’ (MAYO-54//(SEL.29-1-C)NORIN-10/BREVOR) and ‘Anza’ (LERMA-ROJO-64//NORIN-10/BREVOR/3/3*ANDES-ENANO) were used as *Pst* spreader border at the University of California-Davis wheat breeding program and replicated throughout the breeding site. Although natural and strong *Pst* infections occurred regularly in this region (Maccaferri et al., 2015) and no fungicides were applied, a stripe rust nursery located at an edge of the site was inoculated in February (at jointing stage) with a mix of *Pst* spores collected at the University of California–Davis experimental field station during the previous season to ensure a strong disease pressure (Cobo et al., 2018; Dang et al., 2022). The variable distance (0-500m) of ‘DS6301’ and ‘Anza’ non-inoculated replications to the inoculated trials produced a natural gradient of the progression of the *Pst* infection across the field. Data collection was performed on two dates, March 25^th^ and April 22^nd^ 2016. Both dates were preceded by approximately two weeks of no rainfall, with a daily maximum temperature of 74°F on March 25^th^ and 75°F on April 22^nd^. Monthly average daily maximum temperatures for March and April were 68°F and 76°F respectively. Both data collection days were characterized by clear skies providing ideal conditions for canopy reflectance collection. A total of 597 leaf samples were scored with associated hyperspectral observations collected on two collection days, 278 samples on March 25^th^ and 319 samples on April 22^nd^, 2016. In addition, on March 25^th^, 313 canopy hyperspectral observations were collected.

### 2.3 Wheat stripe rust scoring

**Figure 2:**
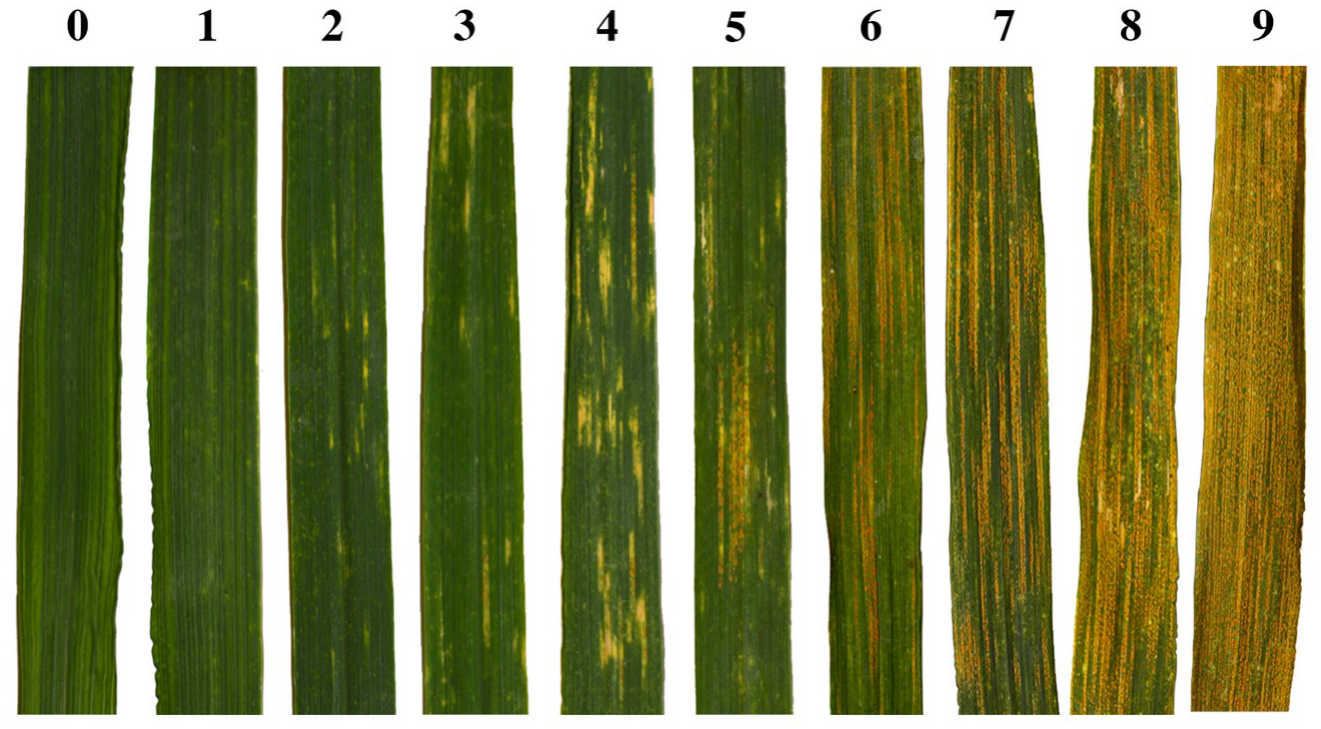
Photographic representations of individual leaves in each of the 10-step classification scale used here for foliar WSR severity assessment. These images were taken from leaves of the highly susceptible ‘DS6301’ line.

Leaves for the leaf-level analysis were sampled from ‘DS6301’ (sown in 1-m rows) and ‘Anza’ (4.4 m^2^ plots), while canopy-level hyperspectral reflectance samples were collected from ‘Anza’ plots only to ensure the sensor field of view was completely composed of the plot canopy. Along with hyperspectral reflectance sampling, we used a modified severity index to estimate the progression of the *Pst* infection as the proportion of the flag leaf affected by rust (Peterson et al., 1948). We modified the commonly used severity index, measured as the percentage of the leaf affected by the disease, and used a 10-step scale to capture early symptoms of infection. Severity class 0 indicates no visible infection symptoms, class 1 shows traces of chlorotic dots, class 2 possess chlorotic spots with traces of sporulation, class 3 shows small stripes with sporulation, and class 4 presents well defined stripes with some sporulation. Severity classes 5-9 all present broad stripes with active sporulation, gradually increasing in percent leaf coverage from 50% (class 5) to 100% (class 9) of disease coverage. **Figure 2** provides example photographic representations of individual leaves in each of the 10-step classification scale used here. Canopy observations were scored using a simplified 5-step scale derived from the more detailed 10-step scale used for individual leaf samples. Consecutive classes are merged together, such that classes (0, 1); (2, 3); (4, 5); (6, 7); and (8, 9) for leaf samples become classes 0, 1, 2, 3, and 4 for canopy observations respectively. Experiments were scored between the heading (Z50) and grain filling (Z80) stages (Zadoks et al., 1974). The *Pst* races detected at the UCD field during the 2016 season, together with their virulence profiles were described previously (Cobo et al., 2018).

### 2.4 Hyperspectral data collection

Visible through shortwave infrared (VSWIR) reflectance spectra were collected with a FieldSpec4 Standard Res field spectroradiometer (Malvern Panalytical, Boulder, CO, USA). This instrument collects radiometrically calibrated radiance observations that are then normalized to reflectance using a standard white reference. The instrument contains three detectors spanning the full 350-2500nm range of the instrument, providing 3nm resolution in the visible through near infrared (VNIR; 350-1000nm) and 10nm resolution at longer wavelengths. Each spectrum sampled is the average of ten spectral samples collected by the system over an approximate one second period. The spectra were then interpolated to 1nm resolution (2151 integer wavelengths) across the full spectral range. Wavelengths less than 450nm and greater than 2400nm were removed due to measurement noise. Model development and analysis was conducted using the reduced spectral range of 450-2400nm (1951 wavebands).

For each leaf sampled on March 25^th^ and April 22^nd^, leaf-level reflectance was measured with the optical fiber attached to a plant probe connected to a leaf clip assembly. This attachment provided a light source, white reference, and black background against which leaf reflectance was collected. On March 25^th^ and April 22^nd^, 278 and 319 leaf-level spectra were collected, respectively. A combined total of 61, 62, 57, 65, 64, 66, 63, 56, 55, and 48 samples were collected for classes 0 through 9 respectively, approximately evenly split between the two days. Each leaf spectrum represents the average of three unique leaf samples assessed to be at the same rust severity class from the same plot. The models developed here include leaf-level models for samples collected on each day, as well as a model developed using all leaf-level data spanning the two collection days.

Immediately following the collection of reflectance spectra all leaf samples were weighed to obtain fresh weight. The samples were then dried for several days in an oven at 40°C until the samples were completely dry. The samples were then weighed again to provide dry weight. Water content was then calculated as the percentage of the fresh weight that was water: (fresh weight – dry weight)/fresh weight * 100.

Canopy-scale reflectance spectra were collected on March 25^th^ using the bare fiber of the spectrometer pointed down onto a wheat plot from a height of approximately one meter above the canopy top. The bare fiber has a 25-degree field of view, producing an approximate 40 cm diameter circular area viewed at the top of the canopy. Measurements were made at the center of each plot, ensuring the entire field of view of the fiber did not extend beyond the plot canopy. A total of 313 canopy spectra were collected across plots spanning the full range of canopy-level severity classes. 67, 55, 70, 54, and 67 samples were collected for classes 1 through 5 respectively. The 1350-1500nm and 1800-1950nm ranges were excluded from the canopy spectra analysis due to noise from atmospheric moisture content in the path of the observation.

### 2.5 Machine learning methodology

Random forests (RF) is a widely utilized machine learning method that determines the classification of each sample from the majority ‘vote’ from an ensemble of decision trees (Breiman, 2001; Ham et al., 2005). This ensemble approach addresses the concern that any single tree might not be optimal due to a random partitioning of the data that results in a bias. This approach likewise improves overall model reliability, particularly in the case of highly collinear features as is often the case with hyperspectral data (Ma et al., 2013; Maxwell et al., 2018). RF has been shown to have superior accuracy and reliability in classifying multispectral data in a suite of case studies relative to other state-of-the-art machine learning techniques (Lawrence and Moran, 2015). In the context of hyperspectral data, RF ensembles require relatively low computational time and demonstrate robustness and high performance relative to other machine learning techniques (Ham et al., 2005; Joelsson et al., 2005), in part due to the ability of RF to handle data characterized by a large number of features and relatively small sample size (Ghamisi et al., 2017).

In addition to the extensive demonstrations of RF performance across disparate problem domains, RF provides valuable analytical tools such as out-of-bag error estimation and feature importance estimation that provide insights on model reliability and the significance of specific spectral features, aiding in the interpretation of the classification results (Li et al., 2023).

Here we utilize random forests for the classification of wheat stripe rust severity at both the leaf and canopy scales, utilizing dimensionality reduction to reduce noise while improving model performance and reliability. We contrast two feature reduction methods, principal component analysis (PCA) and backward feature elimination, which are further detailed in the following section.

For each of the four datasets (March 25^th^ leaf dataset, April 22^nd^ leaf dataset, combined leaf dataset and canopy dataset) the optimal number of PCA components was determined by minimization of the Corrected Akaike Information Criterion (AIC_C_) scores. The average AIC_C_ for a given number of PCA components was calculated from 60 repetitions, using a 20% validation holdout partition of the dataset for PCA models spanning from 1 to 150 components. In each training repetition, random forest hyperparameters were tuned following MATLAB’s hyperparameter optimization scheme for the “fitcensemble” function on a 5-kfold internal cross-validation. We narrowed this optimization to adjust only the number of learning cycles and the learning rate of the model. The number of ensemble trees was set to 100 and bagging was selected for the ensemble aggregation method. Other hyperparameters were left at default values and are the same for all models developed in this study. The AIC_C_ scores for each dataset were fit to a smoothing spline to reduce variance for identification of the optimal number of PCA components that provides the best trade-off between model complexity and performance (i.e. parsimonious model selection). Once the optimal number of components to use for each dataset was determined, the final models were retrained with a 20% validation holdout across 200 repetitions. Holdout data was selected at random for each repetition.

A similar framework was employed for models using backward feature elimination. For each of the four datasets, 100 models were initialized with individual 20% validation holdouts. Each model begins with a feature vector spanning wavelengths from 450-2400nm, 1951 bins for leaf-level models and 1651 bins for canopy-level models. Models iterate through cycles of training and pruning, removing the least significant 10% of features based on feature importance assessment of the trained model to streamline the dataset to the features that are most impactful for prediction. As before, a 5-kfold cross-validated hyperparameter optimization is performed during each training phase. Performance metrics are calculated from the withheld validation data, which is unique to each of the 100 repetitions.

Human labels for each sample were used to train and validate models for rust severity classification. We use two evaluation metrics: accuracy and “off-by-one” accuracy. Accuracy measures the fraction of predicted labels that exactly match the human labels. “Off-by-one” accuracy accounts for human variability by considering a prediction correct if it matches the human label or is within one severity class above or below the human label.

### 2.6 Dimensionality reduction

High-dimensional data such as that produced by spectroscopy provides unique challenges for classification problems due to high data volume, multicollinearity, and a tendency towards overfitting due to the subtle variations in spectral observations (Thenkabail et al., 2014; Ghamisi et al., 2017; Gewali et al., 2018; Burnett et al., 2021; Wang et al., 2021). These challenges are often dealt with by focusing on a limited set of wavelengths (Deng et al., 2023), typically those taken from existing vegetation indices that have demonstrated value in other scenarios (Ashourloo et al., 2014a, b). Problems such as plant disease detection and severity quantification may require unique combinations of wavelengths to optimize model performance (Ashourloo et al., 2014a), ideally taking advantage of relevant information across the full spectral domain (Ashourloo et al., 2016; Schirrmann et al., 2021). High-resolution spectra inherently contain many correlated bands, each potentially providing relevant information that may be redundant with other portions of the spectrum. This redundancy can diminish the performance of classification models by introducing unnecessary complexity and noise (Dormann et al., 2013), while simultaneously incurring the costs of Hughes phenomenon (Li et al., 2023).

Determining a reasonable trade-off for complexity and accuracy is crucial for model simplification. In cases with limited sample sizes, the Corrected Akaike Information Criterion (Equation 1) provides a metric for quantifying model performance as a function of complexity, where *N* is the number of samples and *K* is the number of features (Sugiura, 1978; Akaike, 1998; Portet, 2020).


(1) $$AIC_{c}=MLE-K+\;\frac{2K\left(K+1\right)}{N-K-1}$$

For classification problems with a large number of classes, the maximum likelihood error (MLE) is equivalent to cross entropy, which was calculated here using votes of individual learners (regression trees) within each ensemble to estimate class likelihoods for each data sample (De Boer et al., 2005).

#### 2.6.1 Principal component dimensionality reduction

Dimensionality reduction techniques such as Principal Component Analysis (PCA) are used to preserve data information while reducing dimensionality. PCA aims to produce an orthogonal set of basis vectors that maximally describe the variance in data (Jolliffe, 1990; Jolliffe and Cadima, 2016). This application of PCA centers on maximizing the information content in the input spectra while reducing redundancy, without any influence of a predetermined output or desired classification result. Using this approach a significant reduction of the dimensionality of the input data is possible, greatly enhancing the computational efficiency of ML model development (Herrig Furlanetto et al., 2021). It is important to note however that PCA might overlook fine-scale, yet critical details to the problem of interest, as it is limited by the number of specified components and to patterns in the input data, rather than the classification target (Shafizadeh-Moghadam, 2021; Li et al., 2023).

A key characteristic of PCA is the potential to capture the majority of the variation in a dataset in relatively few components. This allows an approximate reconstruction of the complete spectral observation from only a few components and can aid in associating feature importance as well. **Supplementary Figure S1** (see **Appendix**) displays the relative PCA feature importance determined for the datasets examined here. These scores were transformed by multiplication of the absolute value of the PCA coefficients by feature importance scores to yield importance score spectra. The resulting spectra were summed to yield a single importance spectrum. Through this process, the relative importance of each waveband can be approximated, without directly training on the complete spectral dataset. A similar approach is used in Ginsburg et al. (2015) to rank features on both their PCA embedding and class correlations.

#### 2.6.2 Backward feature elimination

Backward feature elimination is a supervised method that iteratively trains a model and prunes the least relevant features for the task (Speiser et al., 2019). Starting with the entire reflectance spectrum, backward feature elimination methodically removes the least important wavebands, streamlining the dataset to those wavebands that are most important for prediction. The rationale behind selecting only a few wavebands lies in the simplicity and efficiency it offers. We evaluate the optimal selection of features through an iterative backward feature elimination approach, removing the least significant 10% of features based on feature importance assessment in each iteration. We use the built-in feature importance metrics of MATLAB’s Classification Ensembles, which is derived from Gini Importance (Menze et al., 2009). In contrast to PCA, this method removes wavebands from the dataset and focuses on the wavebands that are most relevant for prediction. This difference may make the results of feature elimination more meaningful for the development of vegetative indices and low-cost multispectral instruments for managing wheat stripe rust (Liu et al., 2016).

## 3 Results

### 3.1 Model complexity and dimensionality reduction

**Figure 3:**
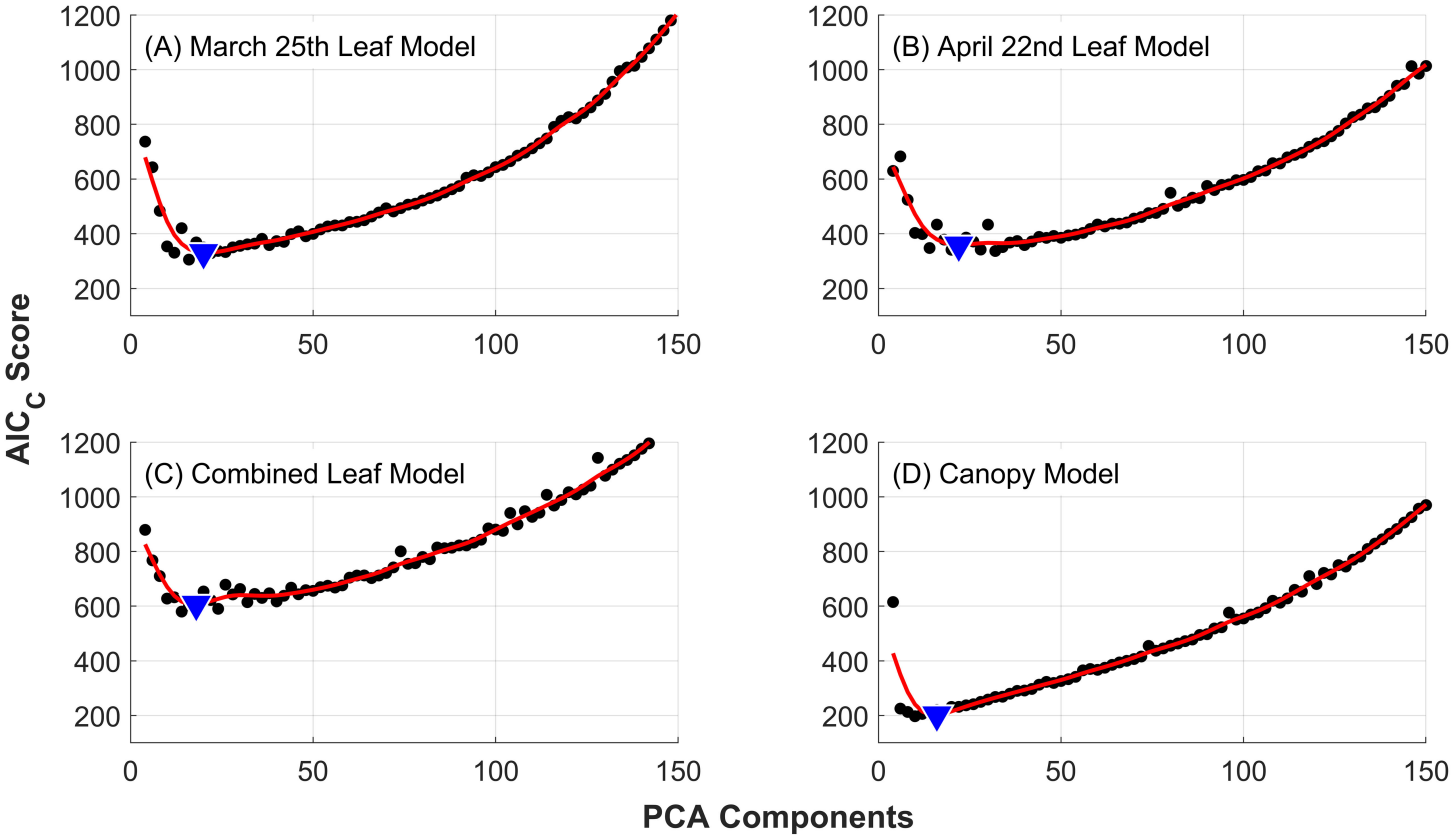
Corrected AIC curves against the number of PCA components used for dimensionality reduction. Individual points (black dots) are the average AIC_C_ of 60 independent models for each number of PCA components evaluated. Fitting splines (red lines) were used to find the minimum AIC_C_ (blue triangles), which determined the optimal number of components to use in the development of the final random forest models. Results are presented for the 10-class severity scale used for leaves: **(A)** March 25 dataset, **(B)** April 22 dataset and **(C)** combined leaf dataset; and **(D)** the 5-class severity scale used for canopy-scale observations.

The results of applying PCA dimensionality reduction to the four datasets are presented in **Figure 3**. The red lines represent smoothing splines fit to the average AIC_C_ scores found for models using from 1 to 150 PCA components. The minimum AIC_C_ values define the optimal number of PCA components used in the development of the final models for each dataset, and were found to be 20, 22, 18, and 16 for the March 25^th^ leaf model, April 22^nd^ leaf model, combined leaf model and canopy model, respectively. The corresponding optimal number of features resulting from feature elimination are 9, 7, 11, and 9 respectively (see **Table 1**).

**Table 1:** Optimal wavelengths retained in the parsimonious models selected using backward feature elimination.

Dataset	Optimal Number of Wavelengths	Optimal Wavelengths [nm]
Canopy	9	454, 643, 668, 680, 690, 702, 758, 766, 782
Combined	11	457, 501, 561, 596, 625, 676, 697, 703, 719, 1425, 1471
March	9	452, 515, 613, 632, 653, 680, 694, 709, 726
April	7	686, 697, 701, 705, 772, 1410, 1470

Wavelengths are presented in numerical order, not in the order of importance for model prediction. The optimal number of wavelengths is determined from minimum AIC_C_ (see **Figure 3**).

### 3.2 PCA classification accuracy

In evaluating the effectiveness of hyperspectral data for classifying WSR severity, we developed four random forest models. Three of these models focused on leaf-level observations and utilized data collected on March 22^nd^ and April 25^th^, as well as the combined leaf dataset from both dates. The fourth model analyzed canopy-level observations from March 22^nd^, which exhibited distinct spectral characteristics compared to the leaf-level data. The results presented here are the average performance of the 200 unique models developed for each dataset, specifically on the 20% of validation data held out during each repetition.

**Figure 4:**
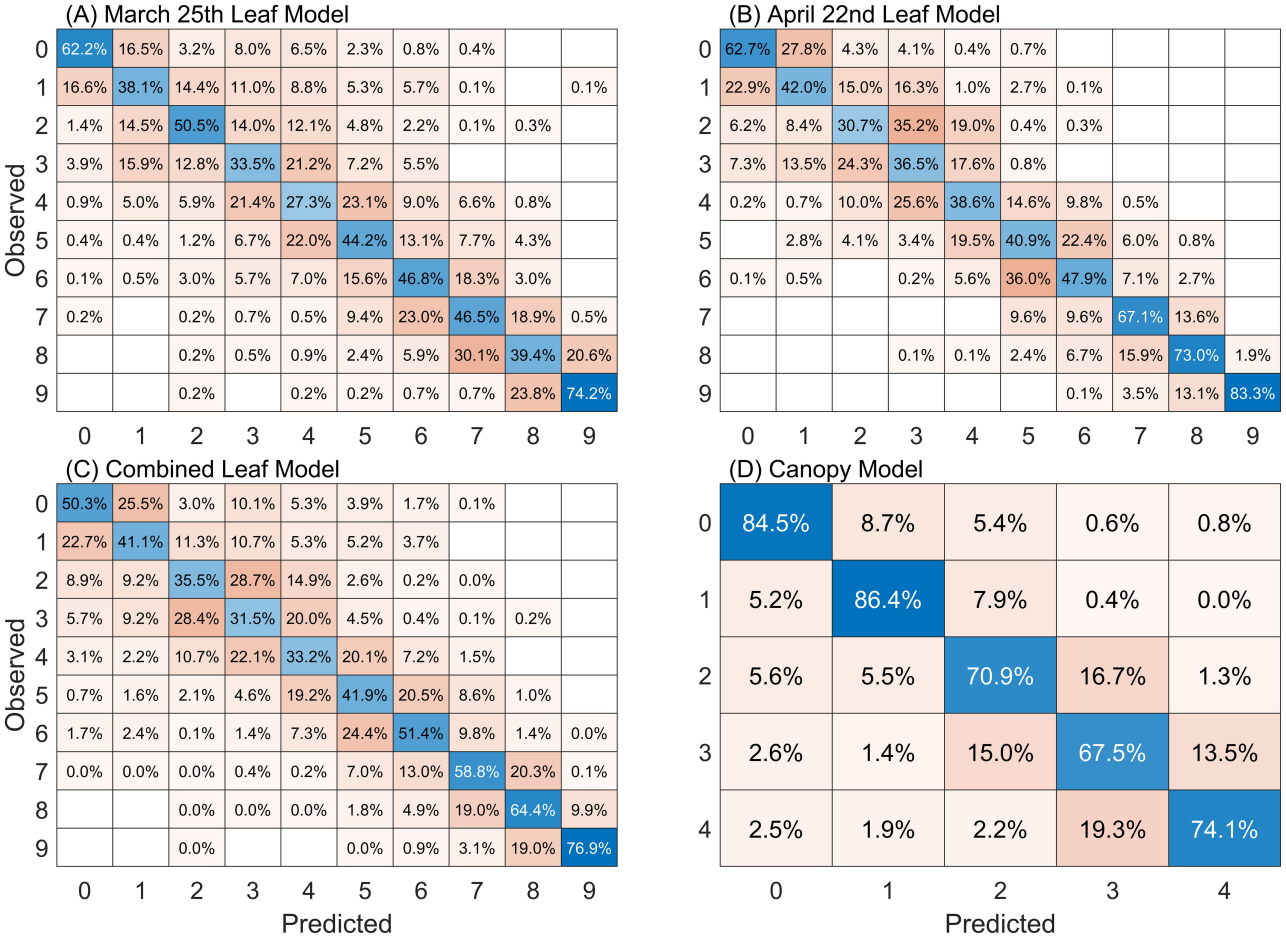
Confusion matrices for RF models following PCA dimensionality reduction. Cell values are the percentage of classifications made for each class. Classification results presented here are for the 20% of data held out for validation, averaged over the 200 model repetitions performed for each dataset. Diagonal (blue) cells show the percentage of accurate wheat stripe rust severity classifications made for each class. Results are presented for the 10-class severity scale used for leaves: **(A)** March 25 dataset, **(B)** April 22 dataset and **(C)** combined leaf dataset; and **(D)** the 5-class severity scale used for canopy-scale observations.

Confusion matrices describing the predictive accuracy of each of the four models on the held-out validation data are presented in **Figure 4**. The leaf-level models for March, April and the combined leaf-level dataset exhibit overall accuracies of 45%, 52%, and 48%, respectively. Similar to early results from Franke and Menz (2007) we find that the presence of fungal spores become easier to detect over time as the symptoms become more pronounced.

We note that for all models and all classes the predicted class is correct more often than an estimation for any single erroneous class. An exception is observed in the April leaf model’s class 2. Observations labeled as class 2 are more frequently predicted as class 3 (35.2%) rather than class 2 (30.7%). We also note that the largest percentage of class mis-predictions occur for classes off-by-one, i.e. that differ from the correct class by one higher or lower severity class. This is true in all instances except for a small number of cases. This suggests that in addition to inherent error that may exist in the RF models that human error in class identification in the field may play an important role in these small errors in class identification. Previous studies have resolved this by reducing the granularity of their classification indices to improve class distinction (Shafi et al., 2023), often using three to four categories that include descriptions such as “asymptomatic”, “pre-symptomatic”, “highly symptomatic”, etc. Here we maintain the original class structure that represents the state-of-the-art in breeding assessments but use an additional “off-by-one” metric, which considers a classification as correct if it falls within one class of the expert human label. Applying this metric, the accuracies for the March, April, and combined leaf-level models improve significantly to 79%, 86%, and 82%, respectively. This approach provides a more realistic assessment of the models’ performance relative to the ground truth observations.

The canopy-level model achieves an overall 78% accuracy and a 96% accuracy using the off-by-one metric. One aspect of this improved performance relative to the leaf-level models is the use of five classes when applying expert human labels in the field for canopy-scale observations, relative to the ten classes used for the leaf-level observations. Similar to the leaf-level models, the canopy-level model exhibited the largest number of misclassifications in classes adjacent to the true class of an observation.

### 3.3 Severity class representation

**Figure 5:**
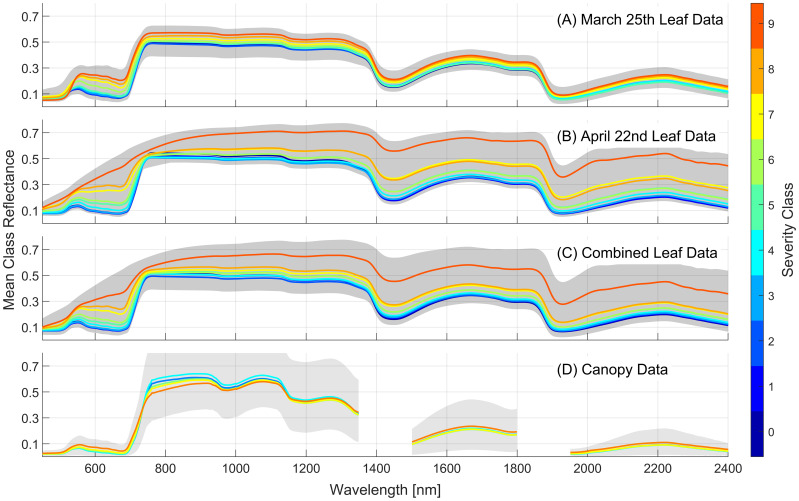
Mean reflectance spectra for each severity class for the two foliar datasets that use 10 severity classes **(A, B)**, the combined foliar dataset **(C)** and the canopy-scale dataset using 5 severity classes **(D)**. Severity class of 0 indicates no infection. The gray regions show the full range of reflectance observed for each dataset.

The mean spectra for each class for each of the foliar datasets and the canopy-scale spectra are presented in **Figure 5**. Generally, an increase in WSR severity class results in increased reflectance across the full 450–2400nm spectral range for the leaf samples. Some variation in the mean reflectance for each severity class can be seen in the two leaf-level datasets collected approximately one month apart. The April 22^nd^ dataset shows larger increases in reflectance in the visible range as severity increases, relative to the data collected on March 25^th^. The highest severity classes in the April 22^nd^ data show higher reflectance in the red portion of the spectrum, and a reduced red-edge transition, perhaps due to increased severity of disease symptoms during this latter data collection period and the onset of necrosis by this date. For canopy-level data the mean spectra show more subtle variations across the 5 severity classes. There is a similar increase in reflectance as severity increases in the visible, but this trend reverses itself in the near-infrared portion of the spectrum. Despite these more subtle variations in reflectance the canopy-scale models showed strong predictive performance across the five severity classes (**Figure 4**).

**Figure 6:**
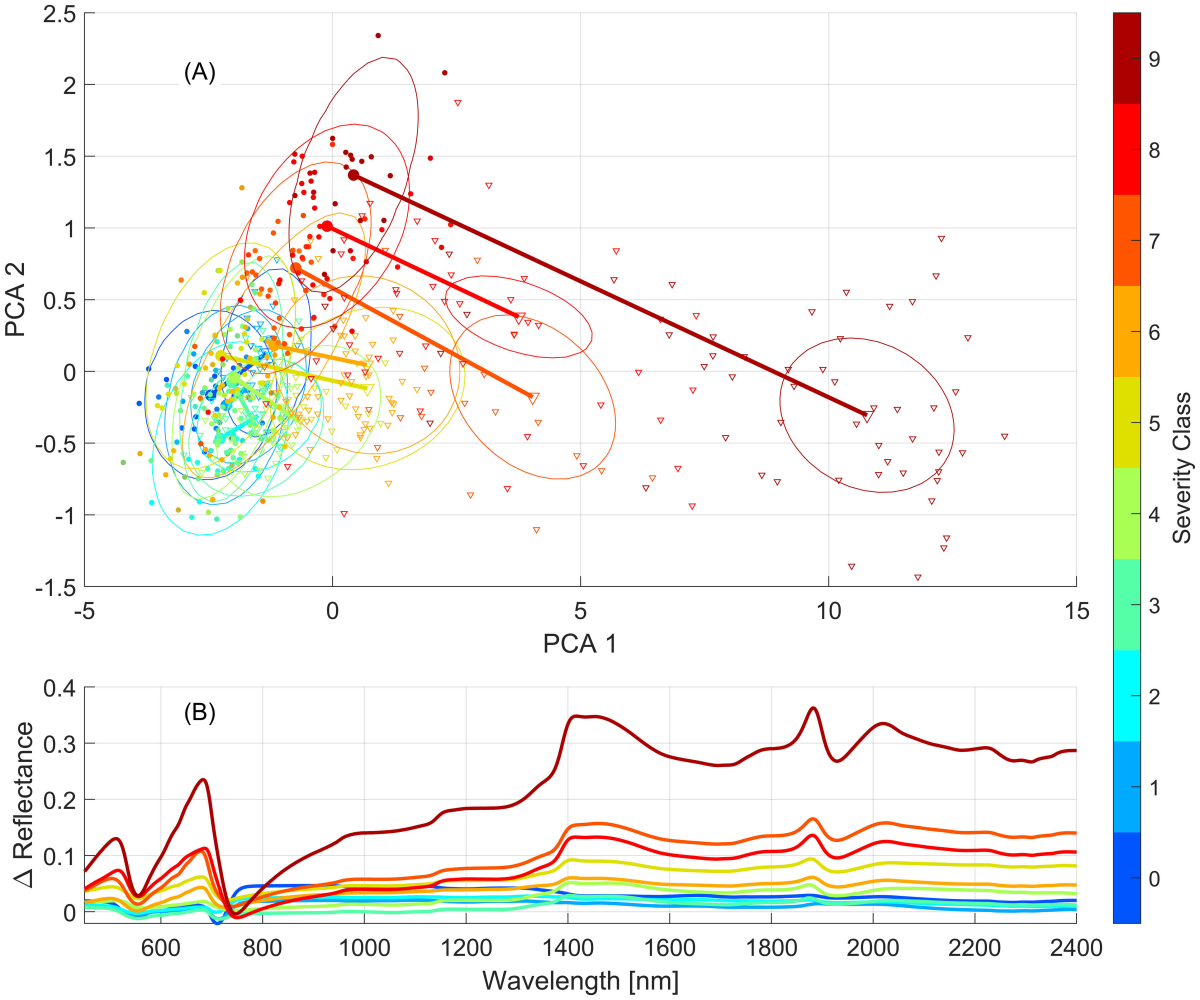
Differences between mean severity class spectra observed on March 25^th^ and April 22^nd^. **(A)** PCA projected distance between the means of classes of March (circles) and April (triangles) reflectance spectra. Ovals are centered on class means and their size is proportional to the spread of points within the class. **(B)** Difference between the mean reflectance spectrum of each class for the two leaf datasets (April – March).

**Figure 6** emphasizes the difference between the March 25^th^ and April 22^nd^ human labelling practices, along with different stages of the disease and characteristics of the lesions produced (orange fungal tissue vs. necrotic tissue). The trend toward higher reflectance in the April 22^nd^ data is apparent with higher severity classes showing more pronounced differences with the March data. Up to severity class 5, there is a significant degree of overlap between the respective classes of March and April, indicating a reasonable similarity between them. From the first two PCA components, classes 7, 8, and 9 show both an increased difference between the two dates as well as an increased variance within class labelling relative to the lower classes. These changes are seen in **Figure 6B** which shows the mean reflectance difference between identical classes for the two leaf collection dates. These differences highlight the variability in human labelling and point to the need for objective and repeatable approaches to quantify severity, particularly in programs targeting the development of resistant germplasm. These differences in foliar scoring between the two dates could be expected to have a confounding effect on the performance of the model developed for the combined leaf dataset, relative to the performance of the models developed for each collection date, but in general this was not found to be the case (**Figure 4**).

**Figure 7:**
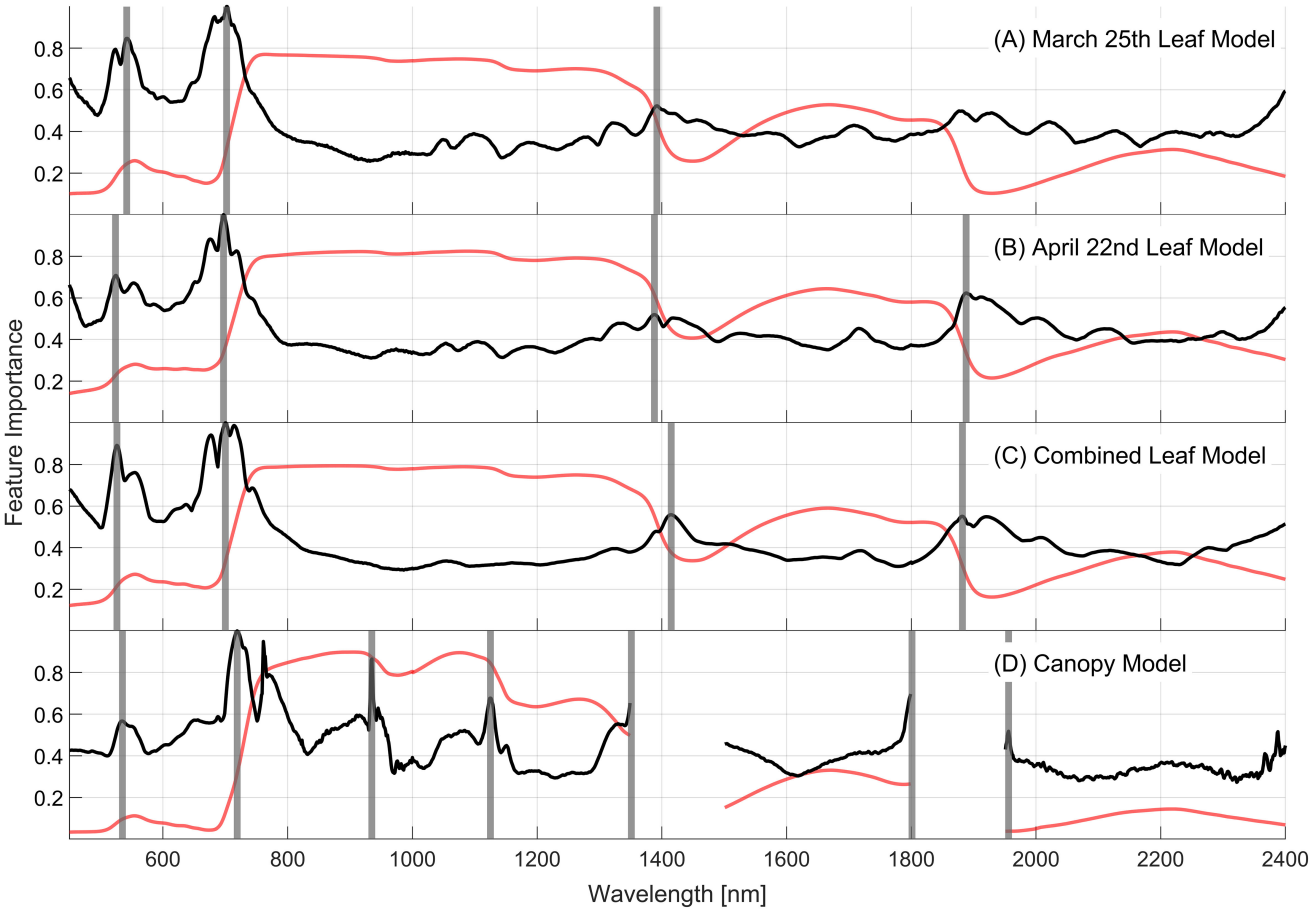
Relative feature importance (black lines) projected across the full spectral range for the final models using PCA dimensionality reduction for each of the **(A)** March, **(B)** April, and **(C)** combined 10-class leaf datasets, and the **(D)** 5-class canopy dataset. The average spectral reflectance (red lines) is presented for each dataset for reference. Vertical grey lines indicate regions of importance in the spectra.

The feature importance for each model projected onto the spectral (450–2400nm) space is presented in **Figure 7**. This measure, derived from the final ensemble of decision trees, shows the impact of each wavelength on the model’s prediction by accumulating the impacts of each PCA component of the final model at each wavelength. The leaf-level models (**Figures 7A–C**) exhibit similar feature importance profiles, with notable peaks at approximately 520, 700, 1400, and 1900nm (vertical grey lines). The April 22^nd^ leaf-level model, however, shows less importance at 520nm and more at 1900nm relative to the March 25^th^ model, perhaps due to changes in pigment and water contents as the plants aged. The combined leaf-level model’s importance profile combines elements of importance seen in the individual models, with lower variability across the 800-2400nm range.

In general, the canopy-level model shares these regions of spectral importance with the leaf-level models but includes new regions of importance at approximately 920 and 1100nm that are not evident in the leaf-level models. The importance peaks located at 1350, 1800, and 1950nm occur at the edges of the regions removed from the analysis due to influences of atmospheric water content. Due to the removal of adjacent wavelengths, these important wavelengths are those that contain information on plant water content, which is likely the reason that the regions around 1400 and 1900nm are important for the leaf-level models. The symptoms of severity class vary as the plants age as seen in **Figures 5A, B**, **7A, B**. Despite this, the similarities between leaf-level feature importance for the two individual leaf-level models suggest that similar patterns in reflectance are consistent with WSR classes as disease symptoms become more severe.

**Figure 8:**
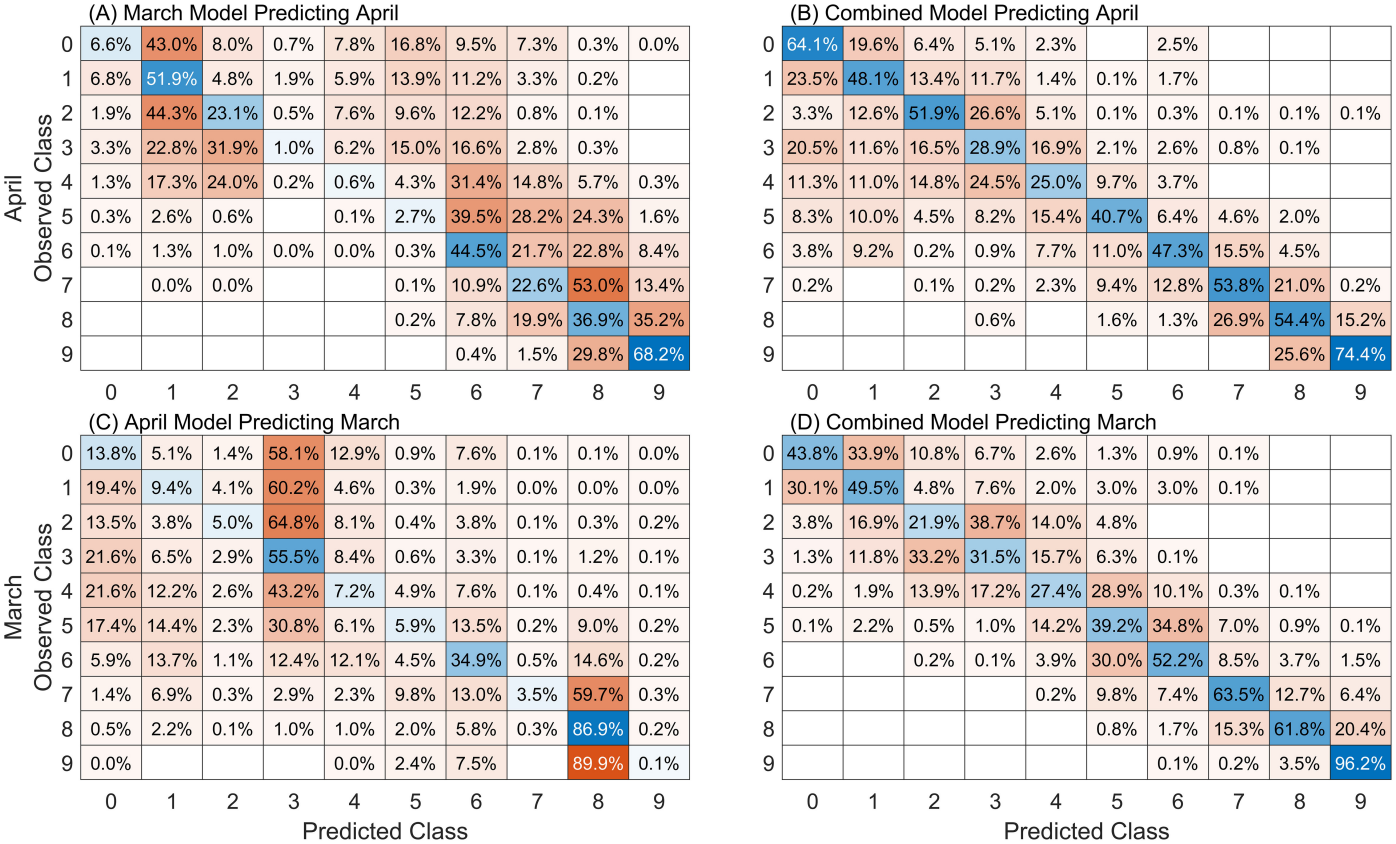
Confusion matrices for leaf models **(A)** developed on March data and applied to April data, **(B)** developed on April data and applied to March data, **(C)** developed on the combined dataset applied to April and **(D)** March data. Cell values are normalized against the number of observed samples in each class. Results are averaged over 200 repetitions of 20% data holdout. Diagonal (blue) cells indicate the fraction of accurate classifications.

An evaluation of leaf-level model performance when applied to datasets for which the model was not specifically trained are presented in **Figure 8**. Confusion matrices for the models developed using March and April data and applied to observations from the other month are presented in **Figures 8A, C**. The performance of the model developed using the combined leaf datasets, and applied to March and April observations is presented in **Figures 8B, D**. These applications allow us to assess the impacts of temporal variability on model performance. When the March model is used to predict April data, performance accuracy drops from 45% (79% off-by-one) to 25% (60% off-by-one). A bias is apparent in the predictions, with very few samples being accurately predicted in the severity classes 3, 4, and 5. In contrast, when the April model is applied to March data it shows a decrease in accuracy from 52% (86% off-by-one) to 22% (54% off-by-one), with a noticeable bias towards overpredicting classes 3 and 8. The combined model, which incorporates data from both periods in model development, demonstrates improved performance. It maintains relatively consistent accuracies of 47% (79% off-by-one) on the March data and 48% (85% off-by-one) on the April data, suggesting that a model trained on a broader range of data can better account for variations due to changes in time of data collection and variability in human labeling on the symptoms and manifestations of wheat stripe rust.

### 3.4 Feature elimination and model parsimony

**Figure 9:**
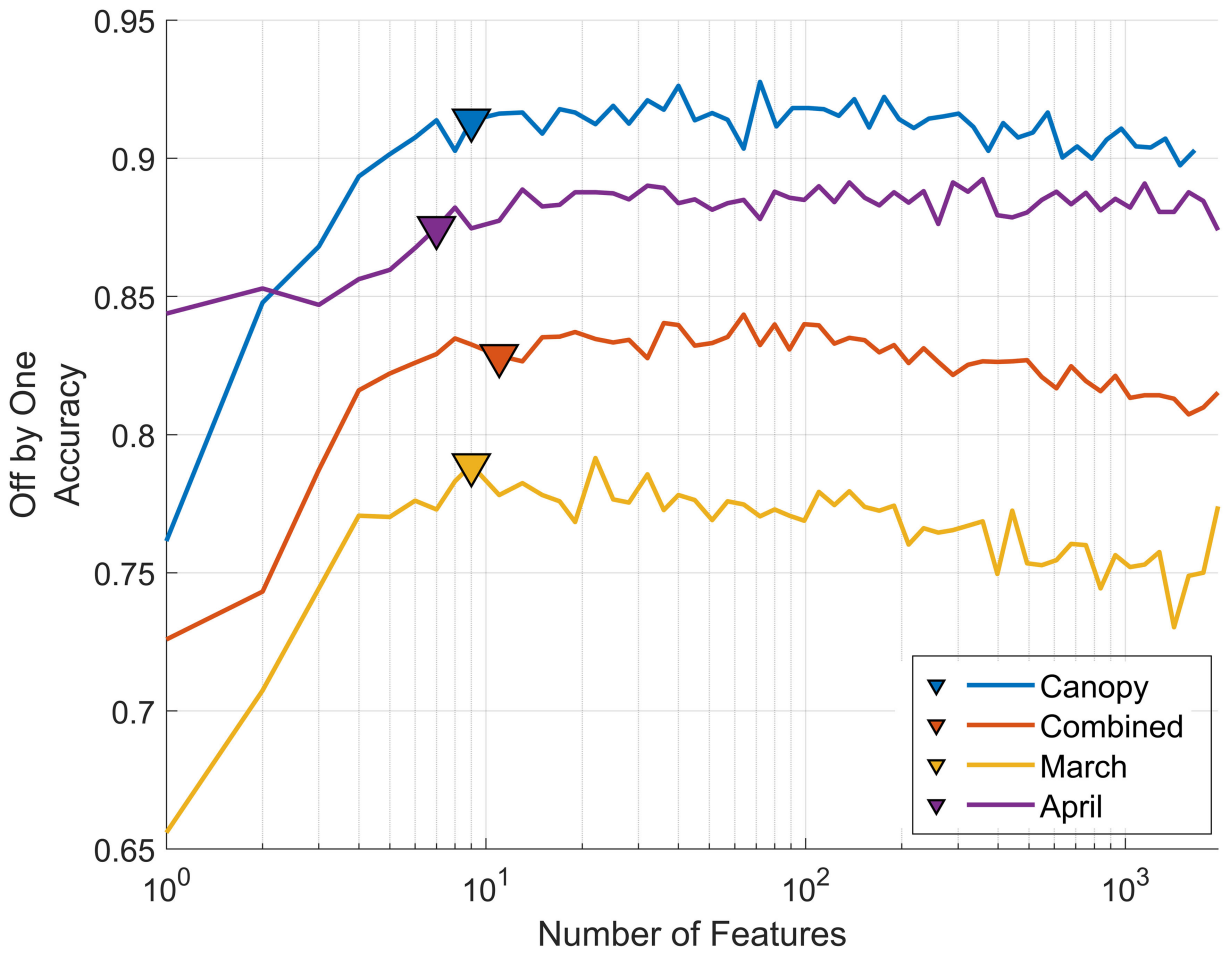
Classification accuracy and feature importance from backward feature elimination. Results are averaged over 100 repetitions using an 80%/20% training-validation split. Mean off-by-one accuracy for each of the four datasets is presented as retained features (wavebands) increase from one to over one thousand. Triangular points indicate the point of minimum *AIC_C_* and define the number of features retained for each of the final models, providing a parsimonious trade-off between model complexity and accuracy.

In addition to the PCA-based dimensionality reduction approach we also implemented a backward feature elimination strategy to select individual wavelengths as model features, rather than the composite values of PCA. This method progressively eliminates the least effective wavebands, allowing us to identify parsimonious models that utilize a reduced set of wavelengths (reduced model complexity) and provide near-optimal model performance. **Figure 9** shows how model accuracy for the four datasets changes as the number of features (wavebands) is increased.

For the case of the canopy model adding relevant features enhances model accuracy from approximately 75% correct off-by-one classifications to over 90% when using 10 features. Beyond this point a performance plateau is reached where additional features do not improve model performance. This behavior is consistent across all models with only slight variations. In each model the most significant wavebands are predominantly between 680-705 nm (see **Table 1**), except for the March leaf-level model, which also includes 450nm and 522nm. These two wavelengths correspond to the regions of peak chlorophyll b absorption (Sauer et al., 1966) and peak reflectance in the green portion of the spectrum, respectively.

In comparison with PCA dimensionality reduction all models exhibit a slight decrease in accuracy when using backward feature elimination, while responses to the off-by-one metric are mixed. Specifically, the combined leaf-level model shows a slight decrease in accuracy from 48% (82% off-by-one) with PCA selection to 45% (83% off-by-one) with backward selection. Similarly, the March and April leaf-level models experience slight drops from 45% (79% off-by-one) to 41% (79% off-by-one) and from 52% (86% off-by-one) to 46% (87% off-by-one), respectively. The ideal number of wavebands was determined using the minimized AIC_C_ score resulting in 9, 7, 11, and 9 wavebands for the March leaf model, April leaf model, combined leaf model and canopy model, respectively. This contrasts with the number of components found with PCA dimensionality reduction at 20, 22, 18, and 16 components respectively, while maintaining a similar level of accuracy.

While these two approaches to reduce the dimensionality of the predictor variables show comparable performance, they differ in how and why they are applied. PCA dimensionality reduction is an unsupervised method which seeks to explain the variance contained in the predictor dataset without consideration of a specific modeling goal. Feature elimination is designed to find the features best suited to the specific modeling task to which it is applied. Speiser et al. (2019) acknowledges that feature elimination methods are well-suited for random forests but are more at risk of overfitting than other feature selection methods. Applications of feature elimination need to consider the specific feature importance metric and how it is used to assess the utility of each feature. This process can be impacted by the general challenges associated with high-dimensional data, particularly sparsity and collinearity.

## 4 Discussion

### 4.1 Feature selection

The two contrasting methods of feature selection and dimensionality reduction (DR) were utilized in this study to provide insights into the most important wavebands for WSR severity quantification, leveraging datasets with high resolution in disease severity scoring and high spectral resolution spanning the full VSWIR region. PCA DR resulted in the identification of regions of importance around 520, 700, 1400, and 1900nm for leaf-level reflectance. The canopy-scale proximal sensing approach also identified 950 and 1100nm as important wavebands. Backwards feature elimination identified narrow regions at 450, 510, 560, 590, 640, 670-700, 720, 760, and 1420nm. These bands include those in the blue (450nm), green (510, 520, 560, and 590nm), and red (670-700nm) as well as the red edge (690-720nm) spectral regions, highlighting the importance of visible color changes associated with fungal growth and possibly changes in pigment contents. When using high spectral resolution data as we have done here (1nm resolution) several neighboring wavelengths may be needed to leverage their relative values, similar to narrow-band vegetation indices (Gupta et al., 2003).

Previous studies have identified a number of wavebands and indices useful in assessing wheat stripe rust incidence and severity. Broadly, wavelengths spanning the green (450–550nm) and red (550–700nm) portions of the spectrum have previously been identified for wheat leaf rust detection (Azadbakht et al., 2019). Several two-band indices commonly used in vegetation remote sensing (i.e. NDVI: [675nm, 800nm], NBNDVI: [680nm, 850nm] and PRI: [531nm, 570nm]) have been shown to be effective for wheat leaf rust assessment (Azadbakht et al., 2019) and detection (Ashourloo et al., 2014b). In a search for optimal combinations of wavebands Deng et al. (2023) identified several wavebands spanning the green, red and red-edge regions of the spectrum as particularly effective for severity assessment, confirming similar findings of Ashourloo et al. (2014a). The findings of these studies support the significance of our identified wavebands for wheat rust assessment. Simultaneously, we identify a few spectral regions that may yield improvements for wheat rust assessment: 640nm, 760nm, 1100nm, 1400nm, and 1950nm.

**Figure 10:**
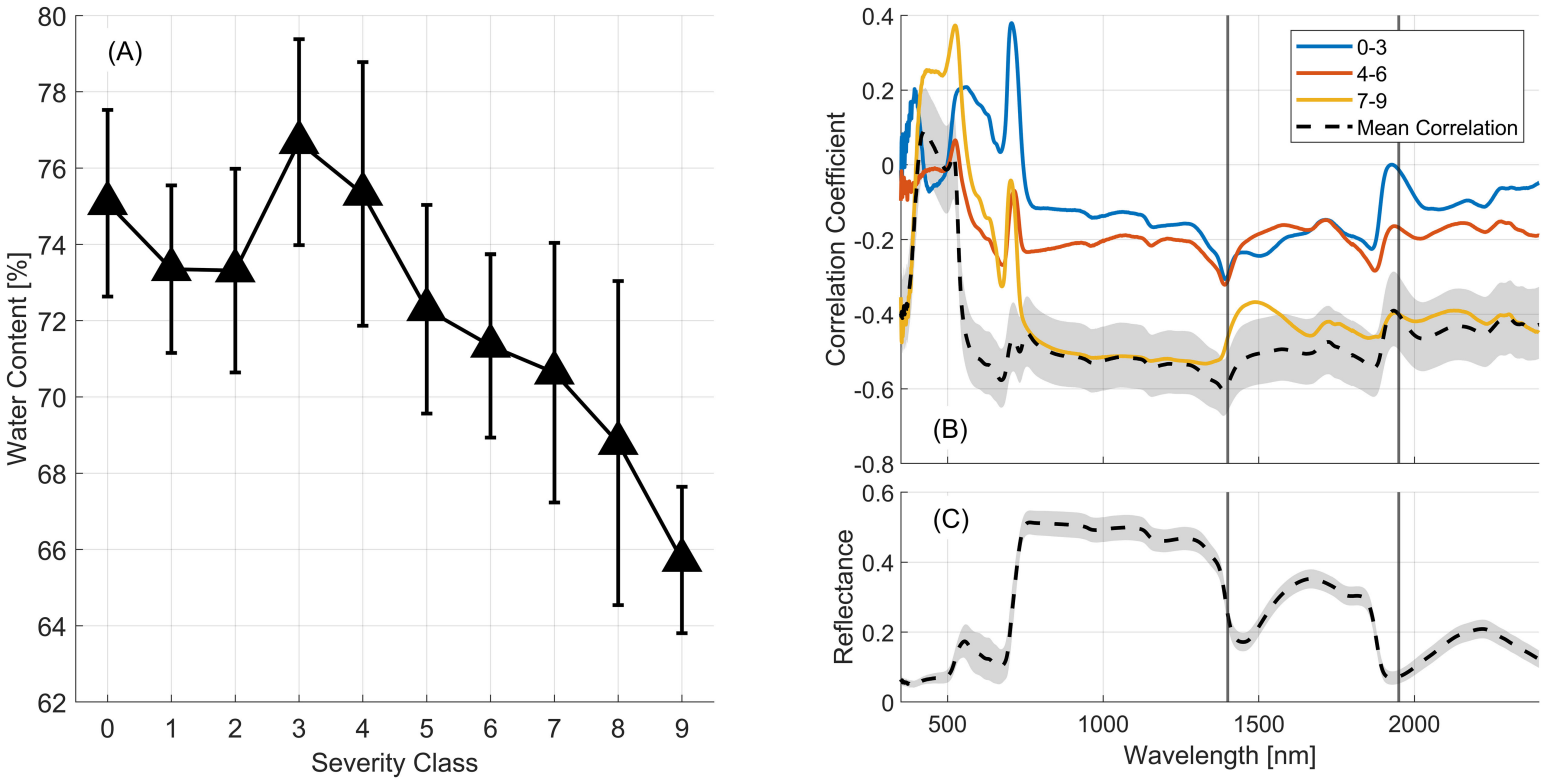
**(A)** The mean (black triangles) and +/- one standard deviation (vertical lines) of leaf water content across severity classes observed for the March 22 dataset. **(B)** Correlation coefficients calculated between the reflectance observed in aggregated severity classes and leaf water content for the March 22^nd^ dataset. The dashed black line shows the correlation over all classes. The gray region indicates the upper and lower bounds of the 95% confidence interval. **(C)** Mean (black line) and standard deviation (gray region) of March 22^nd^ reflectance profiles.

Previous research has demonstrated that increased reflectance around 1400nm and 1950nm correlates with decreased water content and increased rust severity in wheat (Moshou et al., 2004). **Figure 10** supports these findings for the detailed classification used in this study, showing how leaf water content and its influence on reflectance spectra change with severity class for the March 22 dataset. **Figure 10A** shows that as wheat stripe rust severity increases the water content in the wheat leaves decreases, providing support for an area of relative importance in the reflectance spectra in the region where sensitivity to water content exists. We see that at 1400 and 1950nm a significant increase in correlation is evident across all classes (black line). At the most severe stages of infection (yellow line) correlation is increased overall, particularly across the near-infrared region (800-1100nm) and longer wavelengths.

### 4.2 Extension to multi-spectral sensing technologies

Remote sensing applications in agriculture are often constrained by trade-offs related to sensor cost, size and performance attributes such as spectral coverage and resolution. Multispectral sensors have gained popularity and are now widely deployed in agricultural monitoring due to these considerations. These sensors typically utilize on the order of ten waveband ranges, with some sensors offering flexibility in the selection of the wavebands. Previous studies have demonstrated positive results in the use of multispectral imaging for WSR severity assessment (Su et al., 2018; Heidarian Dehkordi et al., 2020). Our results demonstrate that roughly ten narrow wavebands result in parsimonious models that while much simpler than models utilizing the complete spectra available to us offer excellent performance in WSR severity estimation. Here we further simplify the information in our dataset to explore how common multi-spectral instruments would perform for this problem. We use the five waveband ranges of the MicaSense RedEdge-M (MicaSense, Seattle, WA, USA) instrument which spans the visible through near infrared regions with bandwidths ranging from 10 to 40nm: blue (475 ± 10nm), green (560 ± 10nm), red (668 ± 5nm), red edge (717 ± 5nm) and NIR (840 ± 20nm).

To assess the potential of a multispectral instrument for classification tasks across our fine-scale classification system we convolved our hyperspectral reflectance data to the spectral responses of this 5-band sensor, using the waveband ranges above. We note that there are numerous factors (sensitivity, signal to noise ratio, illumination, blur, and pixel uncertainty) which would ordinarily introduce additional noise into a real-world application of this sensor, so these findings should be considered an upper-bound on classification performance.

Using spectra that have been adjusted to represent this much reduced spectral domain we developed RF models using 60 repetitions with an 80%/20% training split. This “multi-spectral” (MS) model produced an average classification accuracy of 58.76% (89.92% off-by-one). In comparison, applying our backwards feature selection methodology on our original 1-nm resolution spectra to derive an optimal five-wavelength model resulted in average classification accuracy of 61.18% (93.58% off-by-one) under the same training conditions. This model used wavelengths at 453nm, 628nm, 689nm, 694nm, and 764nm, confirming the importance of visible wavelengths for this problem, particularly in the red and red-edge regions of the spectrum. Both models produced similar distributions in predictions and misclassifications, suggesting that no significant bias was introduced by the MS model.

In a study using a MS instrument to assess yellow rust severity through unmanned airborne vehicles (UAVs), Su et al. (2018) achieved an accuracy of 89.3% across three severity classes. Spectral indices were derived from the five wavebands and used as features. Due to the nature of how field measurements were conducted, these results are not directly comparable to ours, but generally provide confirmation that sensors providing information in a carefully-chosen small set of wavebands can provide excellent performance and promise to dramatically advance WSR monitoring and management practices.

When applied to our leaf-level data we see an off-by-one accuracy of 74.30%, 87.14%, and 81.36% on the March, April, and Combined leaf datasets for the MS model. When we use backwards feature selection to develop an optimal 5-band model for our leaf data we found that five wavebands can yield off-by-one accuracies of 81.36%, 88.54%, and 84.08% for the March, April, and Combined leaf datasets, respectively. We find that the optimal 5-band model outperforms the MS model for the March dataset for classes 0-4 by approximately 10%. This points to the need to carefully select the specific waveband regions, and spectral resolution, when developing sensors targeting this specific classification problem, particularly when early detection is critical. We found that adding a waveband centered between 640 and 650nm could increase overall off-by-one accuracy to 79.67%, improving early-stage off-by-one accuracy from 64.13% to 79.50% for severity classes 0-4.

## 5 Conclusion

This study focused on the application of hyperspectral reflectance observations to classify wheat stripe rust severity for a 10-class and 5-class scale for leaf-level and canopy-level spectra, respectively. We developed and evaluated four random forest models to assess the ability of machine learning to accurately classify WSR severity across this finely resolved severity classification system. The three leaf-level models (one for each collection date, and one for combined data across both collection dates) exhibited overall accuracies between 45% and 52%. The introduction of an “off-by-one” metric, which considers a classification correct if it falls within one class of the expert human label, provides a more meaningful comparison given the error-prone nature of human classification. This approach realized accuracies between 78% and 82%. This suggests that severity classes have distinct spectral features that become more pronounced as the disease symptoms become more severe. The canopy-level model, with a 5-class system, achieved an overall accuracy of 78%, increasing to 96% using an “off-by-one” assessment. This study provides one of the most rigorous tests of the use of hyperspectral reflectance observations for WSR classification and provides evidence that machine learning and hyperspectral reflectance observation can be leveraged for precision remote monitoring and management of WSR to limit crop damage and to aid in the selection of resilient germplasm in breeding programs.

Analysis of reflectance spectra for severity classes identified both temporal and structural variation in human labelling, complicating the classification problem. Leaf-level data revealed that human labeling can vary over time as observations made at different growth stages may be biased by the progression in disease severity across an experiment, or phenological changes of the plants. Leaf-level and canopy-level (proximal) reflectance classification experiments demonstrated consistency in the important regions of the reflectance spectrum required for accurate class identification.

Overall, feature importance analysis across models indicated that wavelengths in the green, red, and red-edge portions of the spectrum were important for WSR classification, as well as regions associated with variations in plant water content. Commonly deployed multispectral instruments may be adequate for late-stage wheat rust classification. We find that the addition of a single narrowband observation around 640nm has the potential to significantly improve early-stage wheat rust detection in standard (3-5 band) multi-spectral instruments.

We contrasted two approaches to reduce the dimensionality of high-dimensional hyperspectral reflectance data. Methods based on both PCA projection and waveband feature elimination demonstrated that hyperspectral observations can be greatly simplified while maintaining a high degree of classification accuracy. Parsimonious models were identified that required approximately ten wavebands for both leaf and canopy-level data, providing an optimal trade-off between model complexity and performance. This points to the potential to develop multi-spectral sensors specifically for fine-scale classification of WSR for precision treatment and enhancing breeding program evaluations. This study demonstrates the potential of hyperspectral measurements to accurately distinguish and classify WSR severity during the critical early stages of leaf infection for targeted and efficient stripe rust management.

## Data Availability

The raw data supporting the conclusions of this article will be made available by the authors, without undue reservation.
