# Supplementary material for: Non-invasive diagnosis of wheat stripe rust progression using hyperspectral reflectance
Source: Front Plant Sci. 2024 Sep 11;15:1429879. doi: 10.3389/fpls.2024.1429879 (PMC11422131; doi:10.3389/fpls.2024.1429879)
Supplement: Supplementary file 1 [file DataSheet1.docx]

Supplementary Material

Non-Invasive Diagnosis of Wheat Stripe Rust Progression Using Hyperspectral Reflectance

**James F. Cross, Nicolas Cobo, Darren T. Drewry^*^**

*** Correspondence:** Darren T. Drewry: drewry.19@osu.edu

# Supplementary Figures

**
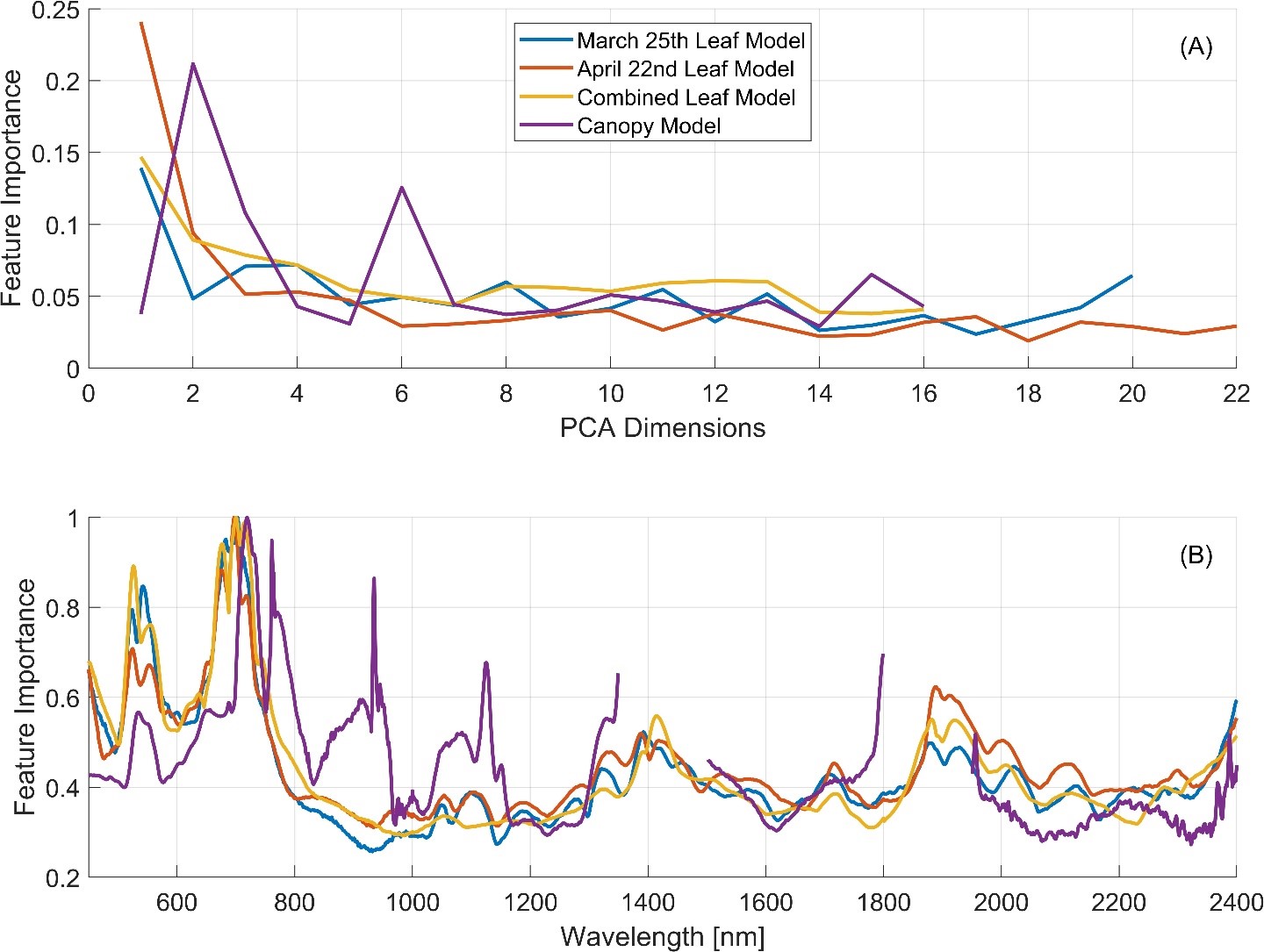
**

**Supplementary Figure S1.** Feature importance for (A) PCA features and (B) projections onto reflectance spectra across the full wavelength range. Feature importance spectra are scaled on [0, 1] for visual uniformity.
